# LGP2 Expression is Enhanced by Interferon Regulatory Factor 3 in Olive Flounder, *Paralichthys olivaceus*

**DOI:** 10.1371/journal.pone.0051522

**Published:** 2012-12-10

**Authors:** Jun-ichi Hikima, Mi-Kyong Yi, Maki Ohtani, Chan Yong Jung, Young Kyu Kim, Ji Young Mun, Young Rim Kim, Haruko Takeyama, Takashi Aoki, Tae Sung Jung

**Affiliations:** 1 Aquatic Biotechnology Center, College of Veterinary Medicine, Gyeongsang National University, Jinju, Gyeongnam, South Korea; 2 Department of Life Science and Medical Bioscience, Waseda University, Shinjuku, Tokyo, Japan; 3 Consolidated Research Institute for Advanced Science and Medical Care (ASMeW) Waseda University, Shinjuku-ku, Tokyo, Japan; INRA, France

## Abstract

In innate immunity, LGP2 (laboratory of genetics and physiology 2) plays a very important role in the production of type I interferon (IFN) through recognition of cytosolic viral RNA. Although viral infection or stimulation with double-strand RNA dramatically induces expression of the LGP2 gene, the underlying transcriptional mechanism has never been studied. Here, we cloned and characterized the 5′-upstream region (−1,337 bp) of the LGP2 gene in olive flounder (*Paralichthys olivaceus*). Numerous canonical motifs for IFN-regulatory factors (IRFs) were found in this region, and reporter assays identified a poly I:C-responsive promoter region (−506 to −398) that regulated LGP2 transcription. Transcriptional activity of the LGP2 promoter was strongly enhanced by IRF3, which bound to IRF3 motif #3 (−480). The LGP2 promoter was also responsive to viral infection *in vitro*. These results suggest that LGP2 transcriptional control is crucially involved to regulated by IRF3 function after viral infection or stimulation with poly I:C.

## Introduction

Innate immunity is responsible for the immediate, acute immune response to pathogens. Pattern-recognition receptors (PRRs), which recognize pathogen-associated molecular patterns (PAMPs), such as viral nucleic acid, peptidoglycan and lipopolysaccharide (LPS), represent the first line of defense and thus play an important role in the innate immune response [1]–[3]. The PRR family includes Toll-like receptors (TLRs) and RIG-I (retinoic acid-induced gene-I)-like receptors (RLRs). TLRs recognize extracellular PAMPs, whereas RLRs detect cytoplasmic PAMPs; both perform crucial roles in the response to viral infections [2], [4].

RLRs, including RIG-I, MDA5 (melanoma differentiation-associated gene 5) and LGP2 (laboratory of genetics and physiology 2/DExH box polypeptide 58), are a class of PRRs known as intracellular RNA sensors that induce type I interferon (IFN) in the innate immune response [5]–[7]. RIG-I detects short double-stranded (ds) RNAs up to 1 kb and 5′-triphosphate single-stranded RNAs (5′ppp-ssRNA), whereas MDA5 recognizes long dsRNAs (>3 kb) and polyinosinic:polycytidylic acid (poly I:C). LGP2 binds to both 5′ppp-ssRNAs and dsRNAs [8]. Generally, RLRs are composed of two N-terminal caspase recruitment domains (CARDs), a DExD/H-box helicase domain and a C-terminal regulatory domain (RD); LGP2 differs in that it does not possess a CARD. The central DExD/H box RNA helicase domain has the capacity to hydrolyze ATP and bind, and possibly unwind, RNA [8]. The RD of RLRs binds to dsRNA, and CARD usually binds to the RD to prevent signal transduction in the absence of viral RNA [9]–[11]. The CARDs of RIG-I and MDA5 interact with the CARD of IPS-1 (IFN-β promoter stimulator-1, also known as MAVS, VISA or Cardif) as an adaptor molecule and initiates downstream signaling through activation of TBK1 (TANK-binding kinase1) and IKK-*i* (inducible IκB kinase) [8]. These kinases phosphorylate interferon regulatory factor (IRF)-3 and −7, which in turn activate transcription of type I IFNs and IFN-inducible genes (ISGs) [12].

While it is clear that RIG-I and MDA5 serve as PRRs for viral PAMPs, a role for LGP2 in antiviral responses is uncertain. Because LGP2 lacks N-terminal CARDs, it is thought to be unable to directly interact with IPS-1, an interaction that may negatively regulate the activated antiviral signaling mediated by RIG-I or MDA5 [11], [13]. Overexpression of LGP2 has been shown to result in decreased IFN production in cells following viral infection or transfection with poly I:C. Furthermore, LGP2-deficient mice exhibit enhanced resistance to viral infection, and embryonic fibroblasts isolated from these mice show increased IFN expression in response to poly I:C. However, positive regulatory roles of LGP2 in RIG-I/MDA5-mediated signaling have also been reported in LGP2-deficient mice, which have lost the ability to synthesize type I IFNs and are unable to intensify efficient antiviral responses against encephalomyocarditis virus infection [14].

Recently, the LGP2 gene was cloned from numerous teleost species, including the olive flounder, *Paralichthys olivaceus* [15]; fugu, *Takifugu rubripes* [16]; Atlantic cod, *Gadus morhua* [17]; rainbow trout, *Oncorhynchus mykiss* [18]; and grass carp, *Ctenopharyngodon idella* [19]. Expression of the LGP2 gene in these species is dramatically increased by viral infection or poly I:C stimulation [15], [18], suggesting that LGP2 transcriptional responses to the pathogen or dsRNA stimuli are important for RLR-dependent signaling. However, transcriptional control of the LGP2 gene, including its regulation by promoter or enhancer elements, has not been analyzed in any vertebrate.

In a previous study [15], we identified the LGP2 gene from olive flounder (*Paralichthys olivaceus*) and characterized its antiviral function. In this study, we cloned the 5′-upstream region of the olive flounder LGP2 gene, which was predicted to serve as a transcriptional control region, and analyzed its transcriptional activity using transient reporter assays. It is anticipated that understanding molecular mechanisms underlying the antiviral response, such as those described here, will lead to our comprehension of the basis of comparative innate immunology and the development of methods for preventing outbreaks of infectious diseases in fish farms.

## Materials and Methods

### Sequence Analysis of the Olive Flounder LGP2 Promoter

The nucleotide sequence of the 5′-upstream region (approximately 1.34 kb) of the olive flounder LGP2 gene was determined by a gene-walking method using specific primers, listed in Table 1. Transcription factor binding motifs in this region were identified using MatInspector (http://www.genomatix.de/en/index.html) software in conjunction with a zebrafish database provided in the software. Nucleotide sequences of LGP2 upstream regions in other vertebrates, namely green spotted puffer (*Tetraodon nigroviridis*), Japanese medaka (*Oryzias latipes*) and humans (*Homo sapiens*), obtained from the Ensembl genome browser (http://www.ensembl.org/index.html), were also analyzed by MatInspector.

**Table 1:** Oligonucleotide sequences.

Genes (GenBank ID)	Purpose (%*)		Name	Oligonucleotide sequence (5′→3′)
LGP2	Real-time PCR (92%)		WCUP-158	GATGATGCAGATGATCCAAGACTACA
(HM070372, HM100666)			WCUP-159	CTCGCTGCTCTAAATCACACCACAT
	Gene walking		WCUP-463	CTTGGCCACATACACAGCAG
	Common reverse primer for reporter vector		WCUP-670	GGCAAGCTTTCAACACATTGTTTAGA
	Forward primers for each reporter vector	LP(-1214)	WCUP-671	GTTACGCGTGTTTCCAACATTGGT
		LP(-1085)	WCUP-672	GTAACGCGTGCATTCATGGCCAAA
		LP(-789)	WCUP-673	GATACGCGTATTCTGCAGATGGAG
		LP(-506)	WCUP-674	CTTACGCGTGTGCATTGTCAGTAT
		LP(-453)	WCUP-1453	GCTACGCGTGACTTGCAGCTGAAAGGAAA
		LP(-396)	WCUP-675	CATACGCGTGTATTTCATGCAACAC
		LP(-51)	WCUP-676	GAAACGCGTAGCTGATGACAACAC
	GFP-expression vector	LP(-506)-GFP	WCUP-1331	GCGGAATTCCCTTGTGCATTGTCAGTATG
			WCUP-1332	CGTAAGCTTTCAACACATTGTTTAGATGGTG
β-actin	Real-time PCR (100%)		WCUP-90	TGATGAAGCCCAGAGCAAGA
(AU050773)			WCUP-91	CTCCATGTCATCATCCCAGTTGGT
Mx	Real-Time PCR (102%)		WCUP-361	AGGCAAAGATTGAAACCATAAAGC
(AB110446)			WCUP-362	CTGTCCTGGGAGTAAACCATCAT
IRF1	Real-time PCR (123%)		WCUP-947	ATGCCTGTGTCCAGGATGA
(AB005883)			WCUP-948	GCTTCCAGGGAATGGAGAA
IRF3	Real-time PCR (100%)		WCUP-651	AGCTGGTGGAGCAGTTCCTA
(GU017417)			WCUP-652	CATCACCTTCAGCCCTCTGT
IRF7	Real-time PCR (94%)		WCUP-902	TAAAATCAATGAGTTCCCCAATG
(GU017419)			WCUP-903	TGCTTGGAGTTGTCCTCTATCAT
MDA5	Real-time PCR (101%)		WCUP-164	TTCACGAGCGACCTCTGGAT
(HQ401014)			WCUP-165	CACTCTATGCCACGGTACACCAT
ISG15	Real-time PCR (95%)		WUCP-257	TCGAGGGATTTACTGGGTTAGG
(AU261156)			WCUP-258	TTTGTCCACTGGGTCACTGATG

* Amplification efficiency.

### Cell Lines

Hirame natural embryo (HINAE) cells, derived from olive flounder embryos [20], were maintained at 20°C in Leibovitz’s L-15 medium (Life Technologies, Carlsbad, CA, USA) supplemented with 10% fetal bovine serum (FBS; Life Technologies), 100 units/ml of penicillin, 100 µg/ml of streptomycin, and 250 ng/ml of amphotericin B (Life Technologies).

### Poly I:C Transfection

HINAE cells were seeded in 12-well plates at a concentration of 1×10^6^ cells/well and cultured at 20°C. Prior to transfection, cells were washed with PBS, and the medium was replaced with Opti-MEM (Life Technologies). Transfections were performed with Lipofectamine 2000 (Life Technologies) according to the manufacturer’s instructions. A mixture of poly I:C (0.1 and 1 µg) and Lipofectamine 2000 was added to each well, and cells were incubated for 6 hours. Thereafter, Opti-MEM containing the poly I:C/Lipofectamine mixture was replaced with fresh L-15 medium, and cells were harvested after incubating for an additional 6, 12, 24 or 48 hours for RNA extraction.

### cDNA Synthesis and Quantitative Real-time Polymerase Chain Reaction Analysis

Total RNA was extracted from HINAE cells using the TRIzol reagent (Life Technologies). cDNA was synthesized from total RNA (1 µg) by reverse-transcription with a High-capacity cDNA Reverse Transcription Kit (Life Technologies), according with the manufacturer’s instructions, after first treating RNA with DNase I (Fermentas, Burlington, ON, Canada) to remove genomic DNA. Quantitative real-time polymerase chain reaction (Q-PCR) was performed using FastStart Universal Probe Master (ROX; Roche, Basal, Switzerland) according to the manufacturer’s instructions. Primers used for Q-PCR were designed with Primer Express software (Life Technologies). The amplification efficiencies of these primer sets were examined as described by the manufacturer (Applied Biosystems/Life Technologies). The sequences and efficiency values of these primers are listed in Table 1. The specificity of the PCR amplification for all primer sets was verified by dissociation curve analysis. The relative expression levels of olive flounder LGP2, MDA5, Mx, ISG15, IRF1, IRF-3, IRF-7, and IFN-I mRNAs were determined by the comparative Ct (2^−ΔΔCt^) method using the olive flounder β-actin gene as an internal reference. Values are presented as means ± standard errors of the mean (SEMs) of three individual experiments (n = 3). The statistical *p*-values were calculated by Student’s *t*-test.

### Construction of Reporter Vector

Six different length DNA fragments of the olive flounder LGP2 gene upstream region were amplified by PCR from BAC clone #62-12L [15] using specific primers (Table 1). These amplified DNA fragments were cloned into *Mlu*I and *Hin*dIII sites of the pGL3 reporter vector (Promega, Madison, WI, USA). These constructs were named pGL3-LP(−1214), pGL3-LP(−1085), pGL3-LP(−789), pGL3-LP(-506), pGL3-LP(−396) and pGL3-LP(−51). The effects of IRFs were evaluated using pGL3-LP(-453), made as described above. All plasmid DNAs were purified by cesium chloride gradient ultracentrifugation and dialyzed against distilled water.

### Luciferase Reporter Assay

Reporter assays were performed using the firefly luciferase reporter pGL3-basic vector (Promega) and *Renilla* luciferase control vector (phRL-SV40), which was used as an internal control to normalize for transfection efficiency. HINAE cells were seeded in 48-well plates at a concentration of 2×10^5^ cells/well and transfected as described above. For each well, 200 ng of pGL3 constructs [pGL3-LP(−1214), pGL3-LP(−1085), pGL3-LP(−789), pGL3-LP(−506), pGL3-LP(−453), pGL3-LP(−396), pGL3-LP(−100) or pGL3-LP(−51)] were mixed with 40 ng of phRL-SV40 and 0.55 µl of Lipofectamine 2000. Any shortfalls in DNA were compensated by adding pUC19 plasmid DNA (Life Technologies). The effect of intracellular poly I:C stimulation was examined by cotransfecting 50 ng poly I:C (1 ng/µl) with the various reporter vectors as described above. To stimulate cells extracellularly, 10 µg poly I:C (50 ng/µl) was added into the cell culture medium 24 hours post-transfection with the reporter vectors.

The effect of IRFs on LGP2 gene expression was evaluated by transfecting HINAE cells, as described above, with 100 ng of pGL3 constructs, 40 ng of phRL-SV40 vector, and 100 ng of pcDNA4-IRF1, pcDNA4-IRF3 or pcDNA4-IRF7. The construction of pcDNA4-IRF1, pcDNA4-IRF3 and pcDNA4-IRF7 encoding olive flounder IRF1, IRF3 and IRF7, respectively, was described in a previous study [21]. To determine the influence of LGP2 promoter activity by LGP2- or MDA5-overexpression, different amounts of LGP2 or MDA5 constructs (0, 2, 20 and 100 ng), pGL3-LP(−506), phRL-SV40 and 50 ng of poly I:C were cotransfected into HINAE cells. Additionally, different amounts of poly I:C constructs (0, 16, 80 and 400 ng), pGL3-LP(−506), phRL-SV40 and 100 ng of LGP2 or MDA5 expression constructs were cotransfected into HINAE cells. HINAE cells were harvested according to the manufacturer’s instruction for the Dual-Luciferase Kit (Promega) using 50 µl of passive lysis buffer (provided in the kit) for each well. The lysates were centrifuged at 900 *× g* for 5 minutes to remove cell debris, and the supernatants were stored at −80°C until use. Firefly and *Renilla* luciferase activities were measured using 10 µl of each supernatant.

### Regulation of GFP Expression by the LGP2 Promoter in HINAE Cells

To confirm that the olive flounder LGP2 promoter also responds to virus infection, we prepared a green fluorescent protein (GFP) expression construct regulated by the LP(−506) promoter using the pkera-GFP/lys plasmid, which had been constructed in a previous study [21], as a backbone. The LP(−506) fragment was amplified with the primers, WCUP-1331 and WCUP-1332 (Table 1), containing sites for the restriction enzymes *Eco*RI and *Bam*HI at the 5′-ends of the respective primers. The PCR products were cloned into the pJET2.1/Blunt vector (Fermentas) for sequencing. The LP(−506) DNA fragment inserted into the pJET2.1 vector was subsequently digested with *Bgl*II and then ligated into the *Bam*HI sites of the pkera-GFP/lys plasmid, replacing the keratin promoter in the previously created pkera-GFP/lys plasmid [22]. The resulting construct was named pLP(-506)-GFP. HINAE cells (2×10^5^ cells/well) were seeded in 8-well Permanox Lab-Tek chamber slides (177445; Nalge NUNC International, Rochester, NY, USA) 1 day before transfection. pLP(−506)-GFP vector (500 ng) was cotransfected with pcDNA4-IRF3 (300 ng) or poly I:C (300 ng) using Lipofectamine 2000 as described above. At 24 and 48 hours post-transfection, GFP expression was observed under a fluorescence microscope. HINAE cells were infected with viral hemorrhagic septicemia virus (VHSV, ATCC no. GNU2005-1) at a multiplicity of infection (MOI) of 1 at 20°C for 1 hour 24 hours after transfection with pLP(-506)-GFP. After washing with PBS, HINAE cells were cultured for 18 hours at 20°C. VHSV-infected HINAE cells were observed under a fluorescence microscope.

## Results

### Expression of LGP2 and Related Genes in Poly I:C-stimulated HINAE Cells

**Figure 1:**
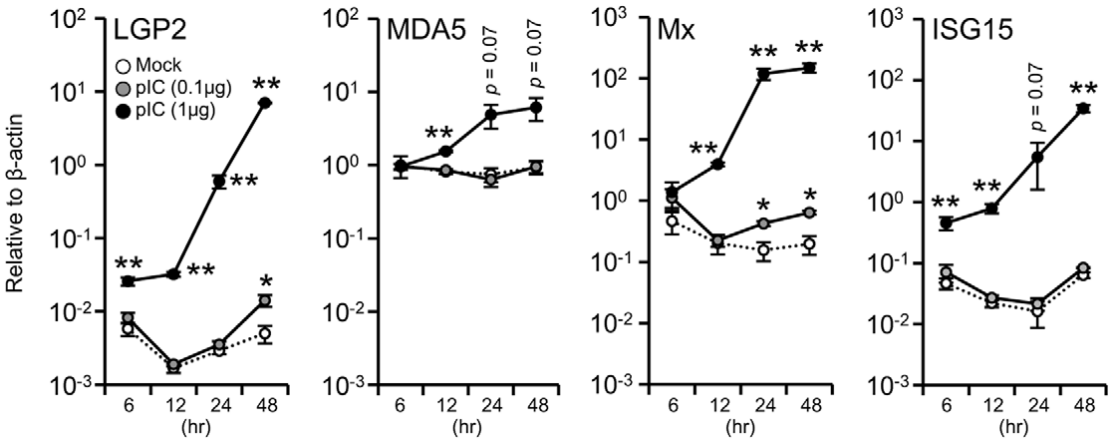
Expression of olive flounder LGP2, MDA5, Mx, and ISG15 mRNAs in HINAE cells stimulated with poly I:C for 6, 12, 24 or 48 hours. Expression levels of LGP2 (**A**), MDA5 (B), Mx (**C**), and ISG15 (**D**) were calculated relative to β-actin mRNA levels. The expression values represent the means ± SEMs of three individual experiments (*p<0.05, **p<0.01, mock control vs. poly I:C-transfected HINAE cell at each time point; Student’s *t*-test).

To evaluate changes in the level of olive flounder LGP2 mRNA following stimulation with dsRNA, we transfected HINAE cells with poly I:C (0.1 and 1 µg) and measured LGP2, MDA5, Mx, and ISG15 mRNAs by Q-PCR. LGP2 mRNA increased logarithmically in cells transfected with 1 µg of poly I:C (Fig. 1). MDA5, Mx and ISG15 genes were also strongly induced in cells transfected with 1 µg of poly I:C. LGP2 transcript levels determined 12 and 48 hours post-stimulation were increased 19.9- and 2,341.7-fold, respectively, compared to mock-treated controls. The relative increases in LGP2 mRNA were much greater than those of MDA5, which was induced by 1.8- and 11.89-fold at 24 and 48 hours, respectively, although the total amount of each mRNA was similar at 48 hours. These results suggest that the transcriptional control of the LGP2 gene is different from that of the MDA5 gene, thus understanding the modulation of the expression of LGP2 and MDA5 genes could be interesting.

### Characterization of the Predicted Transcription Control Region in the 5′-upstream Segment of the Olive Flounder LGP2 Gene

**Figure 2:**
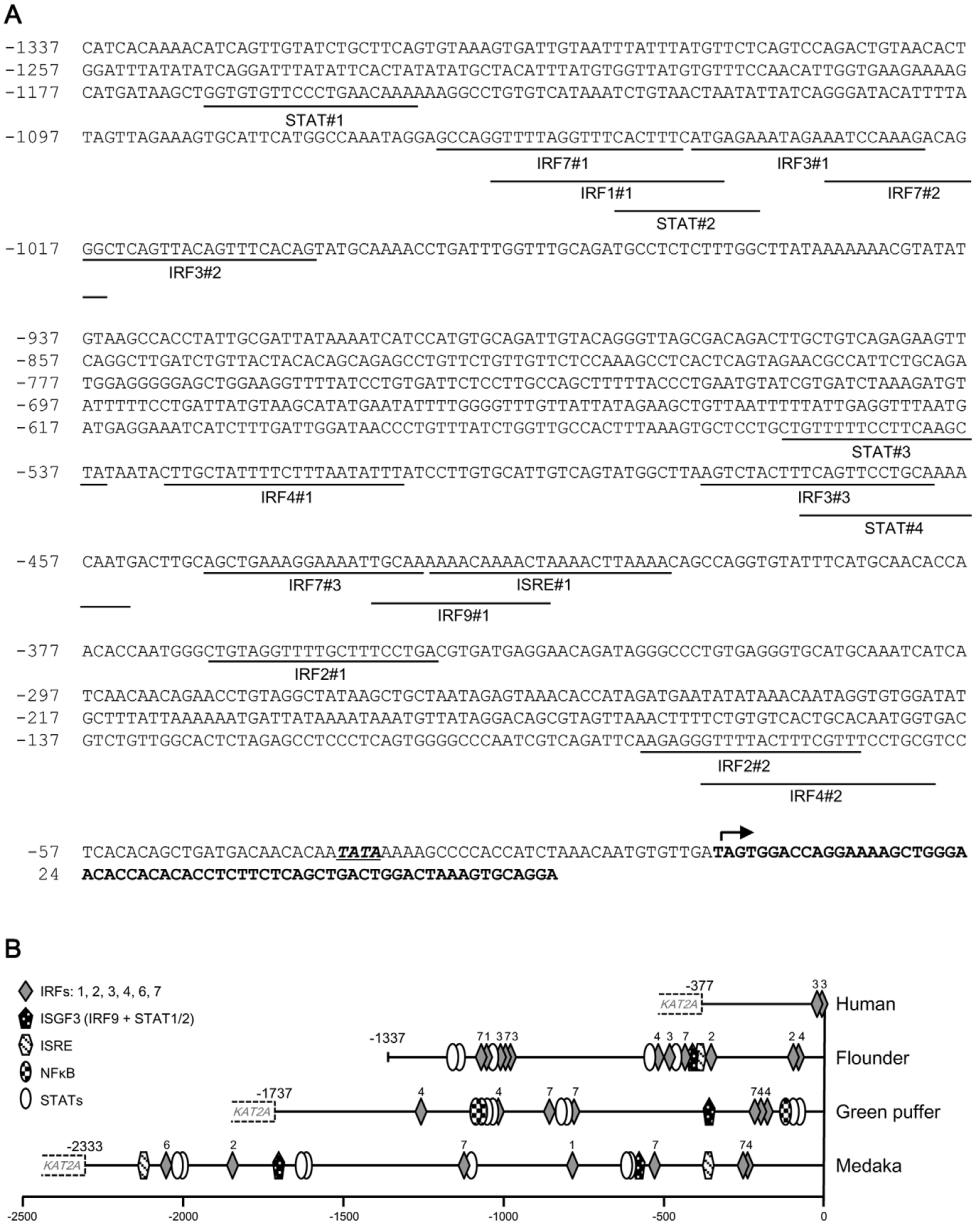
Potential transcription factor binding motifs in the 5′-upstream region of the olive flounder LGP2 gene. (**A**) Nucleotide sequence of the 5′-upstream region is shown with canonical motifs underlined. The transcription initiation site is indicated by an arrow (±0). The TATA box motif is italicized and underlined, and the coding region is in bold. (**B**) Comparison of the 5′-upstream region of the *LGP2* gene in olive flounder (−1,337 to −1) with those of green puffer (Chr. 3∶13152455-13154190), Japanese medaka (Chr. 8∶6135706-6138037, complement), and humans (Chr. 17∶40264652-40265128, complement). The 5′-upstream region between LGP2 and KAT2A genes in humans, green puffer, and Japanese medaka was analyzed using MatInspector software (Genomatix). The schematic diagrams indicate transcription factor canonical motifs, and numbers on the diagrams indicate the types of IRFs.

Numerous canonical motifs that putatively bind transcription factors were identified in the 1,337-bp sequence upstream from the presumptive transcription initiation site [from nucleotide (nt) **-**1,360 to **-**1] using MatInspector software. These include twelve IRF motifs (IRF1, 2, 3, 4, 7, and 9), four STAT (signal transduction and activator of transcription) motifs, and an ISRE (interferon-sensitive response element) motif (Fig. 2A and Table 2). Some canonical sequences of IRF and STAT motifs overlapped each other. A basic TATA box was located at position −34 relative to the transcription initiation site.

**Table 2:** Transcription factor binding sites in the olive flounder LGP2 promoter.

Motif	Coresimilarity*	Start	End	Canonical motif sequence**
IRF1#1	0.87	−1059	−1039	tcatgaaagtGAAAcctaaaa
IRF2#1	0.85	−366	−347	tcaggaaagcAAAAcctacag
IRF2#2	0.85	−87	−67	aaacgaaagtAAAAccctctt
IRF3#1	0.85	−1041	−1021	atgagaaataGAAAtccaaag
IRF3#2	0.85	−996	−1016	ctgtgaaactGTAActgagcc
IRF3#3	0.85	−480	−460	tgcaggaactGAAAgtagact
IRF4#1	0.94	−528	−508	aaatattaaaGAAAatagcaa
IRF4#2	0.94	−81	−61	cgcaggaaacGAAAgtaaaac
IRF7#1	0.86	−1064	−1044	aagtGAAAcctaaaacctggc
IRF7#2	0.86	−1035	−1015	aataGAAAtccaaagacaggg
IRF7#3	0.86	−445	−425	agctGAAAggaaaattgcaaa
ISGF3#1	0.82	−430	−410	tgcaaaaaacAAAActaaaac
ISRE#1	0.81	−424	−404	aaacaaaactAAAActtaaaa
STAT#1	0.87	−1165	−1147	tttgttcagGGAAcacacc
STAT#2	0.87	−553	−535	tagcttgaaGGAAaaacag
STAT#3	0.87	−471	−453	tgttttgcaGGAActgaaa
STAT1#1	0.77	−471	−453	tcagttcctGCAAaacaat
STAT5#1	0.89	−1165	−1147	tgtgTTCCctgaacaaaaa
STAT6#1	0.84	−1049	−1030	ctatTTCTcatgaaagtga
STAT6#1	0.84	−1049	−1030	cactTTCAtgagaaataga

* Core similarity: the element denotes the match of an element to the canonical motif as calculated by MatInspector (TRANSFAC-based analysis).

** Capital letters indicate the core region of the motif sequence.

A synteny analysis of human, medaka, zebrafish, and green spotted puffer genomic databases revealed conservation of neighboring genes between humans and teleosts, specifically showing that the 5′-upstream region of the LGP2 gene was coupled with the KAT2A (lysine acetyltransferase 2A, or GCN5L2) gene (data not shown; also described in [16]). We next analyzed the nucleotide sequence between LGP2 and KAT2A genes in humans, medaka and Tetraodon for canonical motifs using MatInspector software and compared these predicted promoter regions with that for the olive founder upstream region (Fig. 2B). Interestingly, the length of the segment between LGP2 and KAT2A genes was only 377 bp in humans but was much longer (>1.3 kb) in teleosts (Fig. 2B). Only two IRF3 motifs were found in humans, whereas various transcription factor binding motifs, including IRFs, ISREs and binding sites for nuclear factor-kappaB (NF-κB) and STATs were found in teleosts. However, no conserved combination of canonical motifs was found in these sequences.

### Poly I:C Transcriptional Responsive Region

**Figure 3:**
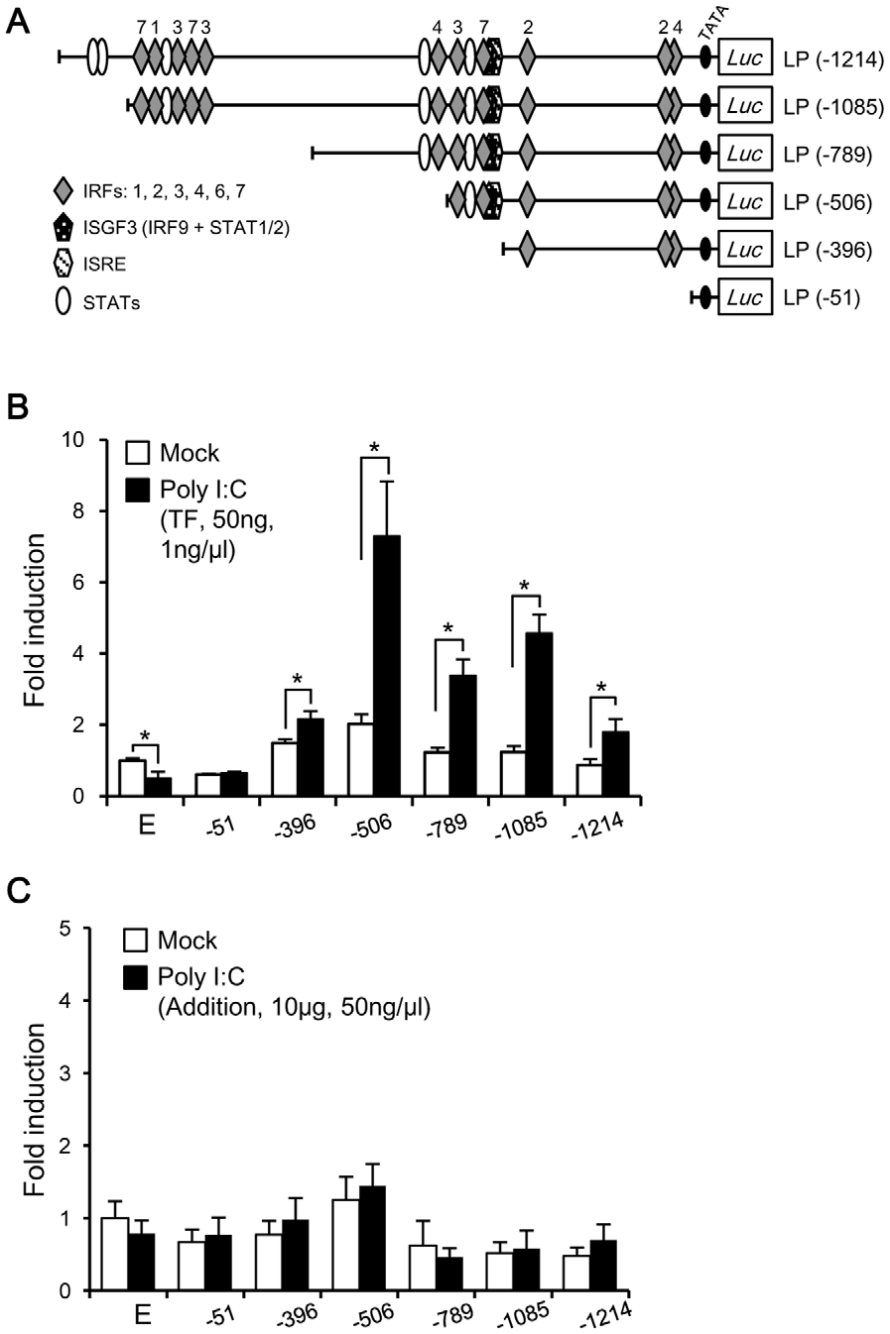
Transcriptional activity of the 5′-upstream region of the olive flounder LGP2 gene in HINAE cells. (**A**) Schematic structure of the olive flounder LGP2 reporter constructs, pGL3-LP(−1271), pGL3-LP(−1096), pGL3-LP(−796), pGL3-LP(−507), pGL3-LP(−400), and pGL3-LP(−51). The schematic diagrams indicate transcription factor canonical motifs, and numbers on the diagrams indicate the types of IRFs. Luciferase activity of reporter constructs transfected into HINAE cells stimulated by poly I:C-transfection (**B**) or poly I:C-extracellular addition to the medium (**C**) was measured 48 hours post-transfection. Luciferase activity is expressed relative to the activity in empty vector-transfected (mock) cells. The activity values represent the means ± SEMs of three individual transfections (*p<0.05, mock vs. poly I:C-transfected cells; Student’s *t*-test).

To study transcriptional activation of the olive flounder LGP2 upstream region by poly I:C, we cotransfected HINAE cells with poly I:C and promoter-luciferase reporter constructs containing six different lengths of this region, and assayed cell extracts for luciferase assay (Fig. 3A). The transcriptional activities of LP(−1214), LP(−1085), LP(−789), LP(−506), and LP(−396) in poly I:C-transfected HINAE cells were significantly higher than those in mock-transfected (control) HINAE cells; the shortest construct, LP(−51), showed no increase in transcriptional activity compared to the mock control (Fig. 3B). The LP(-506) construct exhibited the greatest induction of transcriptional activity by poly I:C stimulation. This result indicates that the region −506 to −51 upstream from LGP2 probably contains the *cis*-regulating element, which mainly enhances the transcription of the olive flounder LGP2 gene. In the LP(−506) construct, the region from −506 to −396 nt was capable of responding to poly I:C-stimulation. Furthermore, the more distal regions from −1,214 to −506, while retaining responsiveness to poly I:C (Fig. 3B), also likely contain repressive elements that dampen the transcriptional response to poly I:C. In contrast, the transcriptional activity in HINAE cells stimulated externally by the addition of 10 µg poly I:C was not induced significantly in all of the promoter regions (Fig. 3C).

### Enhancement of LGP2 Promoter Transcriptional Activity by IRF3

**Figure 4:**
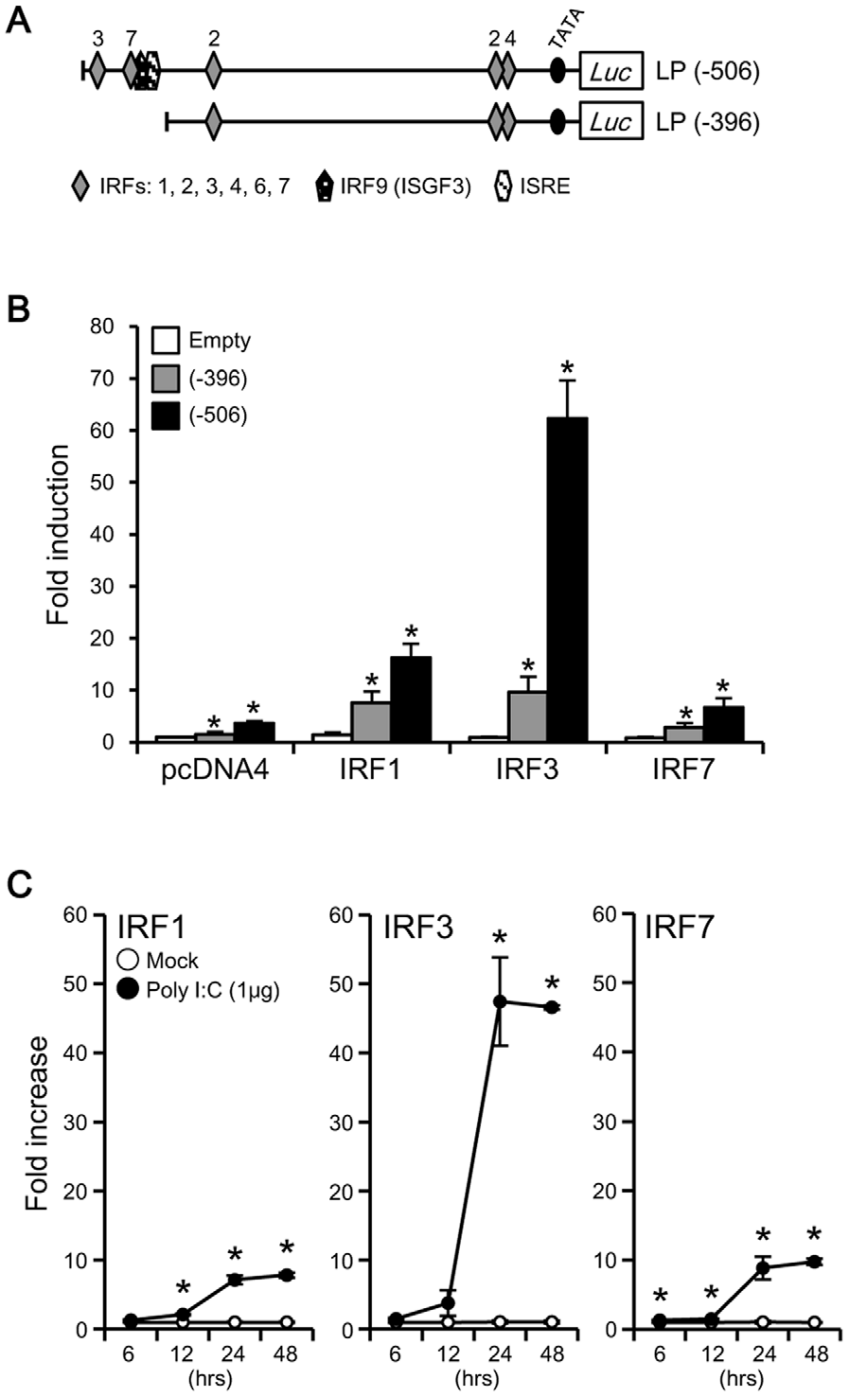
Enhancement of olive flounder LGP2 promoter transcriptional activity by IRF3 in HINAE cells. (**A**) Schematic structure of the reporter constructs, pGL3-LP(−506) and pGL3-LP(−396). The schematic diagrams indicate the canonical motifs, and numbers on the diagrams indicate the types of IRFs. (**B**) Luciferase activity in IRF-cotransfected HINAE cells was measured 48 hours post-transfection. Activity is expressed relative to that of cells transfected with pGL3 and pcDNA4 empty vectors. (**C**) Expression of olive flounder IRF1, IRF3, and IRF7 mRNAs in HINAE cells stimulated with poly I:C for 6, 12, 24 or 48 hours. IRF1, IRF3, and IRF7 expression levels were calculated as fold increase in mRNA levels, normalized to β-actin values, between poly I:C-transfected HINAE cells and mock controls at each time point (6, 12, 24, and 48 hours). Luciferase activity and expression values represent means ± SEMs of three individual experiments (*p<0.01, pGL3-empty vs. pcDNA4-transfected cells [in B], and mock control vs. poly I:C-transfected HINAE cell at each time point [in C]; Student’s *t*-test).

Because the poly I:C-responsive region from −506 to −396 nt in LP(−506) contained many canonical motifs of IRFs, including IRF3, IRF7 and IRSE (Fig. 3), the transcriptional activity of this LGP2 proximal promoter region was examined in IRF-overexpressing HINAE cells transfected with expression vectors pcDNA4-IRF1, pcDNA4-IRF3, or pcDNA4-IRF7. Among the three IRF-overexpressing cells, those coexpressing IRF3 and the LP(−506) reporter showed the strongest activity, exhibiting a 66-fold increase compared to mock-transfected controls (Fig. 4B). In contrast, cells coexpressing IRF3 and LP(−396), which does not contain the presumptive poly I:C-responsive region, showed a dramatic decrease in transcriptional activity. Overexpression of IRF1 or IRF7 induced a small increase in transcriptional activity in HINAE cells transfected with LP(−506), and an even smaller increase in cells transfected with LP(−396). Consistent with these results, a Q-PCR analysis of IRF1, IRF3, and IRF7 transcript levels in poly I:C-stimulated HINAE cells 24 hours post-stimulation showed that IRF3 was induced to the greatest extent of the three IRFs (Fig. 4C).

**Figure 5:**
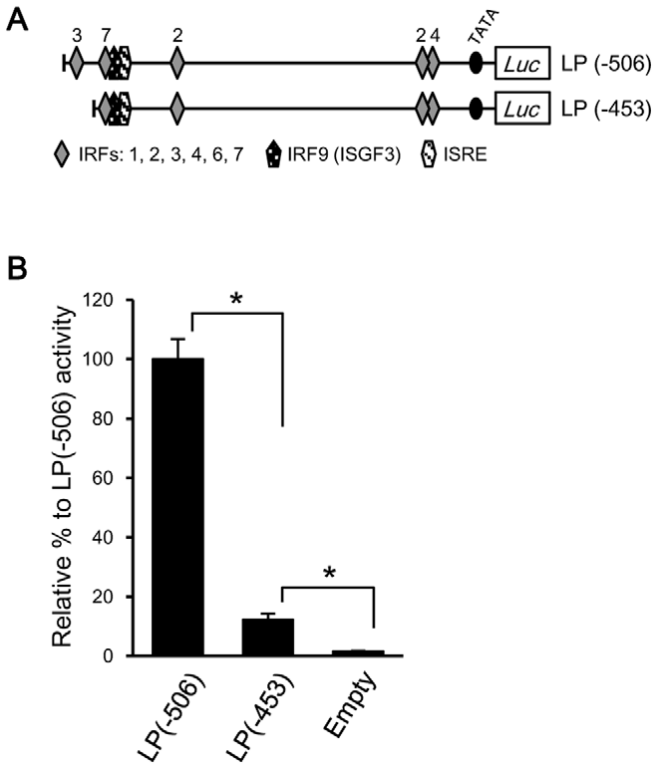
An IRF3 canonical motif in the olive flounder LGP2 promoter region possessing strong transcriptional activity in HINAE cells. (**A**) Schematic structure of the reporter constructs, pGL3-LP(−506) and pGL3-LP(−453). The schematic diagrams indicate the canonical motifs, and numbers on the diagrams indicate the types of IRFs. (**B**) Luciferase activity in IRF3-cotransfected HINAE cells was measured 48 hours after transfection. Activity is shown relative to pGL3-LP(−506) activity in IRF3-cotransfected cells, expressed as a percentage. Activity values represent means ± SEMs of three individual experiments (*p<0.01, pGL3-empty vs. pcDNA4-transfected cells; Student’s *t*-test).

The fact that IRF3 showed the strongest activation of the poly I:C-responsive region in the olive flounder LGP2 LP(−506) promoter construct suggests the involvement of the IRF#3 motif within this region (Fig. 4A). To confirm this, we created and tested another reporter construct, LP(−453), in which the region containing the IRF3#3 motif was deleted (Fig. 5A). Deletion of this IRF3#3-containing region significantly reduced transcriptional activity in the LP(−453) construct, decreasing it by >87.8% compared to that of LP(−506) (Fig. 5B). In fact, the level of LP(−453) activity was similar to that of LP(−398) in Fig. 4B, showing that IRF3 is potentially the most important motif in the poly I:C-responsive region (−506 to −398).

### Effects of the LGP2 LP(−506) Promoter Activity by Different Amounts of LGP2 or MDA5 Overexpression

**Figure 6:**
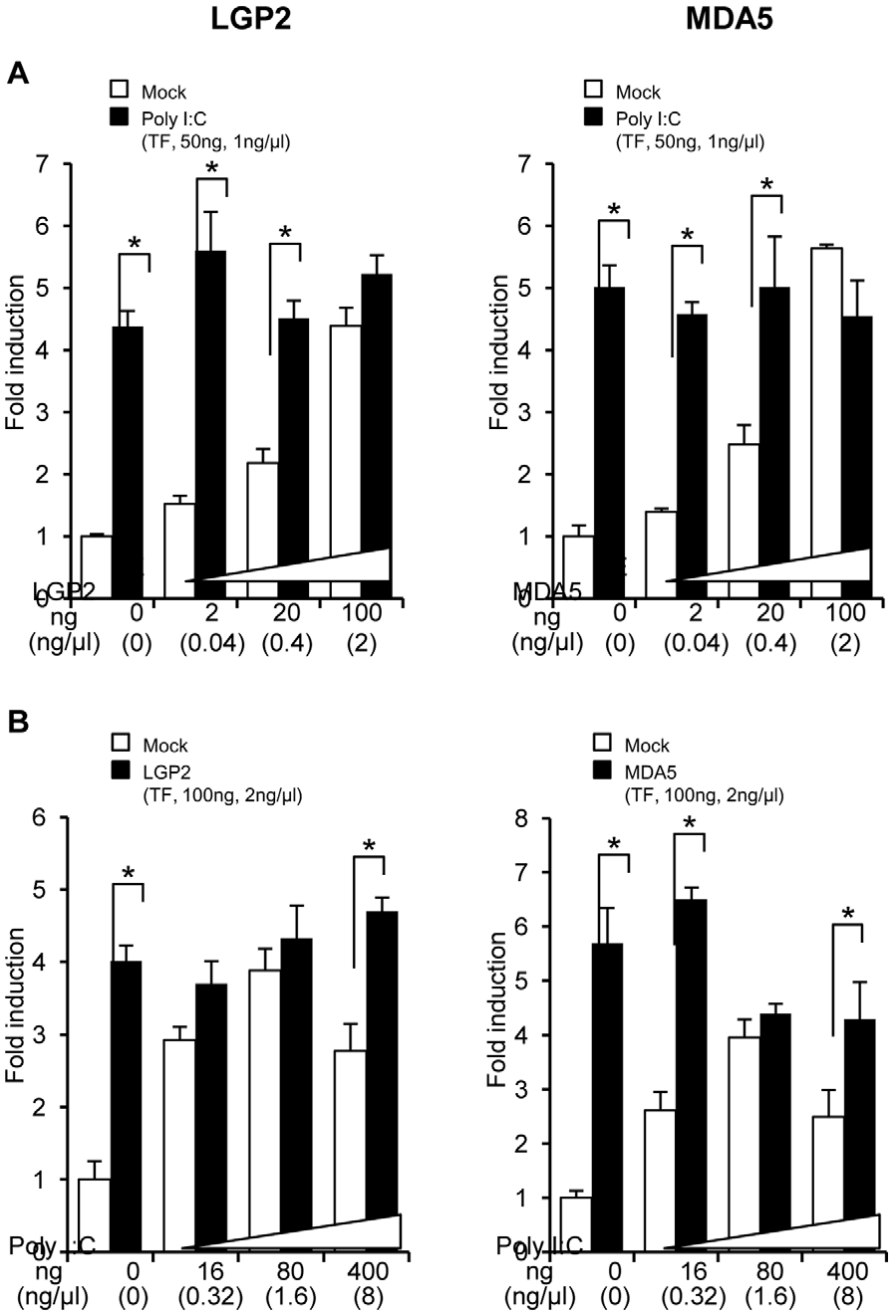
Changes in the transcriptional activity of the LGP2 promoter in LGP2- or MDA5-overexpressing cells stimulated with poly I:C. (**A**) Different amounts of LGP2 or MDA5 constructs (0, 2, 20 and 100 ng) were cotransfected into HINAE cells with 50 ng of poly I:C (transfected). (**B**) Different amounts of poly I:C constructs (0, 16, 80 and 400 ng) were cotransfected into LGP2- or MDA5-overexpressing HINAE cells. Activity is shown relative to pGL3-LP(−506) activity in empty vector cotransfected cells. Activity values represent means ± SEMs of six individual experiments (*p<0.05, pGL3-empty mock control vs. poly I:C- and pcDNA4-transfected cells; Student’s *t*-test). Abbreviation: TF, transfection.

In order to observe changes in the transcriptional activity of the LGP2 promoter with respect to differing amounts of RLR expression vectors, the LGP2 promoter activity was measured in HINAE cells transfected with a variety of doses of LGP2, MDA5, or poly I:C (Fig. 6). When the amounts of RLR were increased from 0, 2, 20 and 100 ng, the activity of LGP2 promoter was enhanced in dose-dependent manner (Fig. 6A). However, when those cells were simultaneously stimulated by poly I:C (transfected), the activity of LGP2 promoter did not change in the cells. On the other hand, when amounts of poly I:C (transfected) were increased from 0, 16, 80 and 400 ng, the activity of LGP2 promoter was enhanced in a dose-dependent manner until the concentration reached 80 ng poly I:C, and then the activity decreased by 400 ng poly I:C (Fig. 6B). When the RLRs were simultaneously overexpressed in the poly I:C-transfected cells, the activity of LGP2 promoter did not significantly change although the MDA5-overexpressing cells stimulated by 80 and 400 ng poly I:C were slightly decreased compared to the others.

### Responsiveness of the LGP2 LP(−506) Promoter Construct to VHSV Infection in HINAE Cells

**Figure 7:**
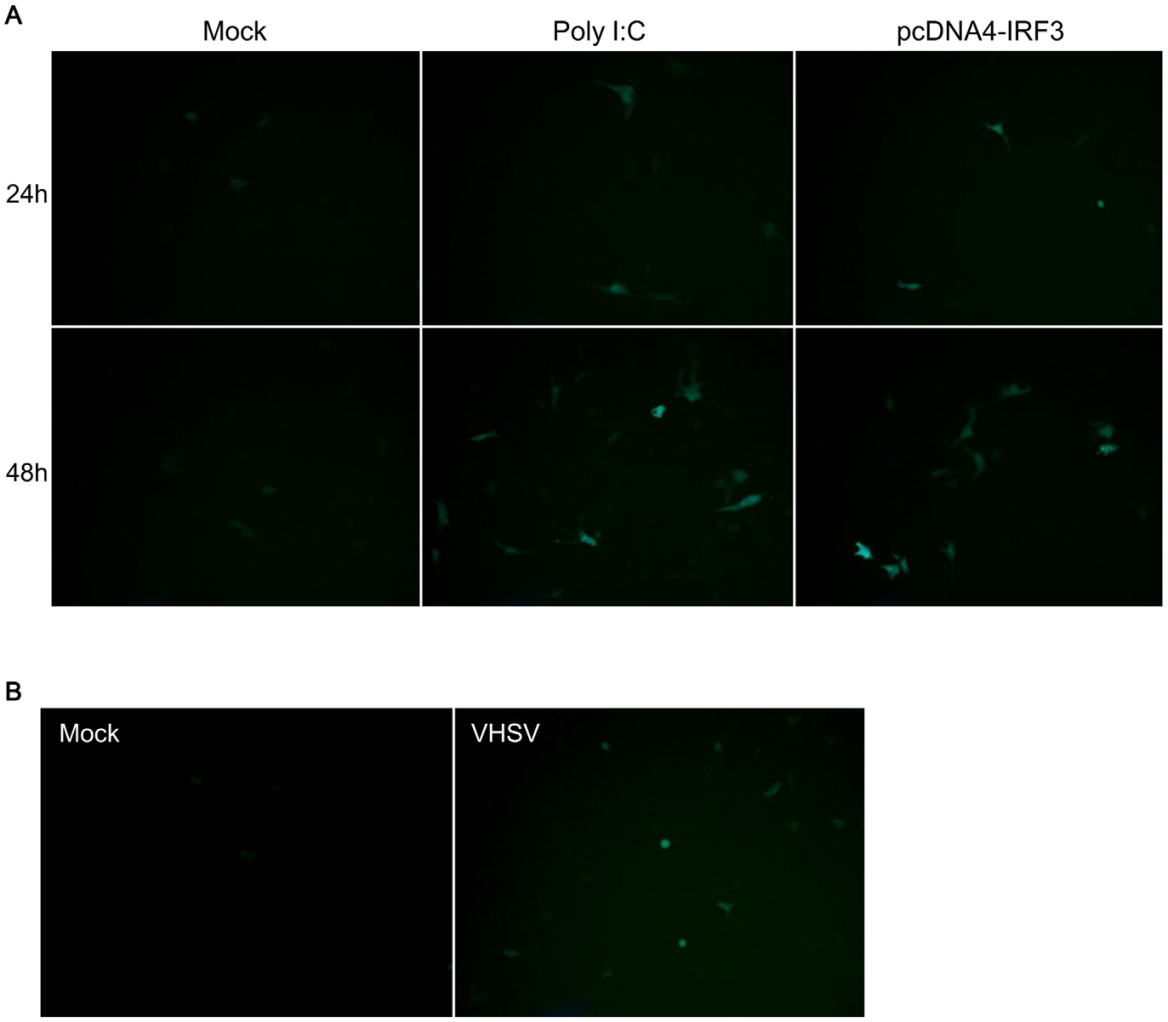
Transcriptional activity of the LGP2 promoter. (**A**) LP(−506) was fused with the open reading frame of the GFP gene and cotransfected with poly I:C (300 ng) or pcDNA4-IRF (300 ng) into HINAE cells. After 24 and 48 hours, the expression of GFP protein was detected by fluorescence microscopy. (**B**) The LP(−506)-GFP vector was transfected into HINAE cells. After 24 hours, cells were infected by VHSV (MOI = 1). After 18 hours, the expression of GFP protein was detected.

To test the responsiveness of the LGP2 LP(−506) promoter region to VHSV infection, we transfected HINAE cells with a pLP(−506)-GFP construct expressing GFP under the control of LP(−506) (Fig. 7A). Preliminary experiments showed that coexpression of poly I:C and IRF3 with pLP(−506)-GFP increased the number of GFP-expressing cells at 48 hours compared to that at 24 hours, demonstrating the effectiveness of our detection system. Notably, infection of HINAE cells with VHSV (MOI = 1) also increased GFP expression at 18 hour post-infection (Fig. 7B), indicating that virus infection enhanced the transcriptional activity of the pLP(−506) promoter. Excessive cell death at 24 hours post-infection due to cytopathic effects prevented us from assaying GFP expression at later time points (data not shown).

## Discussion

In mammals, LGP2 is important in triggering type I IFN production, as evidenced by the fact that LGP2-deficient mice largely lack the ability to produce type I IFN against many virus infections, but not influenza virus [8]. In olive founder and rainbow trout, expression of the LGP2 gene is dramatically induced by poly I:C stimulation and viral infection, and LGP2 has been shown to strongly induce type I IFN gene expression in response to poly I:C transfection [15], [18], [21]. A similar induction pattern is also observed in other fish species, including Atlantic cod, rainbow trout, grass carp, and common carp [18], [19], [23], [24]. However, there is conflicting evidence, for example, in crucian carp LGP2 inhibits poly I:C-induced activation of the zebrafish type I IFN production [24]. This data suggests that LGP2 antiviral function may differ in some species of teleosts, therefore we must be cautious when drawing conclusions.

In this study, we demonstrated a dramatic, dose-dependent induction of olive flounder LGP2 expression in HINAE cells; notably, LGP2 induction was much greater than that of MDA5 (Fig. 1). Interestingly, however, the levels of LGP2 and MDA5 transcripts in the olive founder were similar to each other after induction (Fig. 1). In rainbow trout RTG-2 cells, LGP2 and MDA5 genes are similarly induced by poly I:C transfection [18]. These observations suggests that the transcriptional control of LGP2 and MDA5 may be modulated to balance the numbers of the two molecules, reflecting the fact that LGP2 requires MDA5 for MDA5-mediated viral recognition and recruitment of IPS-1 in the type I IFN induction pathway [2], [8]. Because there is probably no RIG-I in Acanthopterygii fish species such as fugu, medaka and stickleback [16], it is likely that the olive founder genome also lacks the RIG-I gene; thus, the co-modulation of LGP2 and MDA5 gene expression is critically important. Accordingly, the transcriptional regulation of LGP2 expression, which is dramatically upregulated, could play a key role in the activation of type I IFN production. In Fig. 1, the constitutive expression level of LGP2 appears to be much lower than that of MDA5, suggesting that the transcriptional control of LGP2 probably plays an important role in the above-mentioned modulation. Furthermore, although RLR overexpression lead to a dose-dependent enhancement of the LGP2 promoter activity, the highest concentration of poly I:C-stimulation did not increase activity (Fig. 6). It seems that while LGP2 promoter activity is regulated by poly I:C, a large amount may in fact inhibit the activation of the LGP2 promoter. Poly I:C-enhanced LGP2 expression may provide a crucial function for the production of type I IFN, and this evidence could be important to the understanding of the contrasting results observed previously with regard to LGP2 function in teleosts.

Because of the potential importance of this regulatory mechanism, we cloned and characterized the 5′-upstream region of the LGP2 gene in the olive flounder, providing the first information about the transcriptional control of LGP2 gene expression in any animal. Numerous IRF motifs were found in the 5′-upstream region of the LGP2 gene (Fig. 2). This is a typical feature of the 5′-upstream region of the type I IFN gene [21] and type I IFN-inducible genes (*i.e.* Mx and ISG15) [25], [26], which are regulated by RLRs and TLRs in mammals [3], [27]. A comparison of the 5′-upstream region of LGP2 genes in olive founder, humans, medaka, and Tetraodon showed that although the disposition and clustering combinations of motif elements provided no evidence for a conserved promoter structure, the types of canonical motifs, including IRFs and STAT were conserved; notably, IRF motifs were found in all species (Fig. 2B). In this region in the human LGP2 gene, only two IRF3 motifs were found. These observations suggest that the transcription factor IRF is important in regulating LGP2 gene expression.

Results of reporter assays using variable-length constructs of the 5′-upstream region of the olive flounder LGP2 gene showed that transcriptional activity in response to poly I:C stimulation (by transfection) was highest for the LP(−506) construct and notably absent for the LP(−51 construct) (Fig. 3), suggesting that the region from −506 to −51 probably serves as the proximal promoter including the *cis*-regulating element (*i.e.* IRF3#3 site) in enhancing LGP2 gene expression, and the region from −51 to −1 (around −30) probably contains the core enhancer to bind RNA polymerase because of the location of TATA box (Fig. 2). In contrast, extracellular poly I:C stimulation in HINAE cells did not showed any enhancement in transcriptional activity of the LGP2 promoter (Fig. 3C). In a previous study we showed that the LGP2 gene was induced by extracellular poly I:C in olive flounder leukocytes 48 post-stimulation (addition) [15], suggesting that transcriptional control of the LGP2 promoter probably changes with cell-type, for example, immune and non-immune related cells.

Moreover, the activity of the LP(−396) construct was decreased compared to that of LP(−506), highlighting the importance of the −506 to −396 region, where IRF3, IRF7, ISGF3, and IRSE motifs are clustered. The transcriptional activity of the LP(−506) promoter region was strongly induced by IRF3 (Fig. 4B), and IRF3 gene expression was highly induced by poly I:C-stimulation (Fig. 4C). IRF3 also strongly enhances the transcriptional activity of the olive flounder IFN promoter [21], suggesting that IRF3 is tremendously important for signaling cascades activated by poly I:C and further suggesting that LGP2 transcriptional control may be involved in IRF3-regulated pathways, such as those leading to type-I IFN production. Furthermore, the dramatic reduction of transcriptional activity in the LP(−453) construct compared to the LP(−506) construct in TRF3-overexpressing HINAE cells (Fig. 5) suggests that IRF3 probably binds to the IRF3#3 motif, which is a core motif in this promoter. The importance of IRF3 is further reinforced by the presence of significant, albeit reduced, poly I:C-responsiveness in longer constructs [LP(−789), LP(−1085), and LP(−1214)] containing distal IRF3 motifs. The fact that the transcriptional activity of these longer constructs is diminished compared to LP(−506) suggests the presence of repressive elements in these regions.

Experiments using the GFP expression vector pLP(−506)-GFP in HINAE cells showed that poly I:C and IRF3 strongly enhanced the activity of the LGP2 promoter (Fig. 7A). Consistent with the importance of IRF3 in mediating induction of LGP2 in poly I:C-stimulated HINAE cells, described above, expression of the LGP2 gene has been shown to be induced in IRF3-overexpressing CAB cells, a blastula embryonic cell line derived from the crucian carp (*Carassius auratus* L.), another teleost [24]. In humans, two IRF3 motifs are present in the 5′-upstream region of the LGP2 gene (Fig. 2B), suggesting that the human LGP2 promoter could also be regulated by IRF3. Mammalian IRF3 is activated by phosphorylation of TBK1 mediated by TLR or RLR signaling [28]. Zebrafish IRF3 forms a complex with TBK1 and MITA (mediator of IRF3 activation), and this complex is critically involved in activation of the type-I IFN promoter [24]. However, how IRF3 is activated by poly I:C-stimulation in teleosts remains unknown.

The olive flounder LGP2 promoter was activated 18 hours after infection of HINAE cells with VHSV, as evidenced by the induction of GFP expression (Fig. 7B). This result suggests that the olive flounder LGP2 LP(−506) promoter mediates induction of LGP2 expression in response to VHSV infection [15]. Induction of LGP2 gene expression has also been observed in several fish tissues and cells, including grass carp kidney and liver infected with grass carp reovirus, rainbow trout kidney infected with VHSV, and TO cells (a permissive Atlantic salmon head kidney cell line) infected with salmon alphavirus [18], [19].

### Conclusion

The promoter of the olive flounder LGP2 gene was identified in the 5′-upstream region. This promoter, which exhibits a strong transcriptional response to poly I:C-stimulation, contained numerous IRF, ISRE, and STAT motifs. The IRF3#3 motif, located in the poly I:C-responsive region of this promoter, was important for enhancement of the transcriptional activity of the LGP2 promoter by IRF3. The olive flounder LGP2 promoter was activated by both poly I:C-stimulation and VHSV infection, suggesting that the activity of the LGP2 promoter is controlled by IRF3 activated through TLRs or RLRs, which recognize viral ligands. These results provide the first description of the transcriptional control of the LPG2 gene in the entire animal kingdom. Taken together with previous reports, these results indicate that fish IRF3 is closely involved in the transcriptional activation of both type-I IFN and LGP2 promoters, and also plays a very important role in the antiviral innate immune response.

## References

[1] Jun EJ, Kim YK (2009) Activation of innate immune system during viral infection: role of pattern-recognition receptors (PRRs) in viral infection. J Bacteriol Virol 39: 145–157.

[2] Takeuchi O, Akira S (2010) Pattern recognition receptors and inflammation. Cell 140: 805–820. 20303872 10.1016/j.cell.2010.01.022

[3] Loo YM, Gale M Jr (2011) Immune signaling by RIG-I-like receptors. Immunity 34: 680–692. 21616437 10.1016/j.immuni.2011.05.003 PMC3177755

[4] Kumar H, Kawai T, Akira S (2011) Pathogen recognition by the innate immune system. Int Rev Immunol 30: 16–34. 21235323 10.3109/08830185.2010.529976

[5] Kato H, Takeuchi O, Mikamo-Satoh E, Hirai R, Kawai T, et al (2008) Length-dependent recognition of double-stranded ribonucleic acids by retinoic acid-inducible gene-I and melanoma differentiation-associated gene 5. J Exp Med 205: 1601–1610. 18591409 10.1084/jem.20080091 PMC2442638

[6] Schlee M, Roth A, Hornung V, Hagmann CA, Wimmenauer V, et al (2009) Recognition of 5′-triphosphate RNA by RIG-I helicase requires short blunt double-stranded RNA as contained in panhandle of negative-strand virus. Immunity 31: 25–34. 19576794 10.1016/j.immuni.2009.05.008 PMC2824854

[7] Schmidt A, Schwerd T, Hamm W, Hellmuth JC, Cui S, et al (2009) 5′-triphosphate RNA requires base-paired structures to activate antiviral signaling via RIG-I. Proc Natl Acad Sci USA 106: 12067–12072. 19574455 10.1073/pnas.0900971106 PMC2705279

[8] Satoh T, Kato H, Kumagai Y, Yoneyama M, Sato S, et al (2010) LGP2 is a positive regulator of RIG-I- and MDA5-mediated antiviral responses. Proc Natl Acad Sci USA 107: 1512–1517. 20080593 10.1073/pnas.0912986107 PMC2824407

[9] Cui S, Eisenächer K, Kirchhofer A, Brzózka K, Lammens A, et al (2008) The C-terminal regulatory domain is the RNA 59-triphosphate sensor of RIG-I. Mol Cell 29: 169–179. 18243112 10.1016/j.molcel.2007.10.032

[10] Li X, Ranjith-Kumar CT, Brooks MT, Dharmaiah S, Herr AB, et al (2009) The RIG-I-like receptor LGP2 recognizes the termini of double-stranded RNA. J Biol Chem 284: 13881–13891. 19278996 10.1074/jbc.M900818200 PMC2679488

[11] Takahasi K, Kumeta H, Tsuduki N, Narita R, Shigemoto T, et al (2009) Solution structures of cytosolic RNA sensor MDA5 and LGP2 C-terminal domains: identification of the RNA recognition loop in RIG-I-like receptors. J Biol Chem 284: 17465–17474. 19380577 10.1074/jbc.M109.007179 PMC2719387

[12] Kawai T, Takahashi K, Sato S, Coban C, Kumar H, et al (2005) IPS-1, an adaptor triggering RIG-I- and Mda5-mediated type I interferon induction. Nat Immunol 6: 981–988. 16127453 10.1038/ni1243

[13] Rothenfusser S, Goutagny N, DiPerna G, Gong M, Monks BG, et al (2005) The RNA helicase Lgp2 inhibits TLR-independent sensing of viral replication by retinoic acid-inducible gene-I. J Immunol 175: 5260–5268. 16210631 10.4049/jimmunol.175.8.5260

[14] Venkataraman T, Valdes M, Elsby R, Kakuta S, Caceres G, et al (2007) Loss of DExD/H box RNA helicase LGP2 manifests disparate antiviral responses. J Immunol 178: 6444–6455. 17475874 10.4049/jimmunol.178.10.6444

[15] Ohtani M, Hikima J, Kondo H, Hirono I, Jung TS, et al (2010) Evolutional conservation of molecular structure and antiviral function of a viral RNA receptor, LGP2, in Japanese flounder, *Paralichthys olivaceus* . J Immunol 185: 7507–7517. 21098234 10.4049/jimmunol.1001850

[16] Zou J, Chang M, Nie P, Secombes CJ (2009) Origin and evolution of the RIG-I like RNA helicase gene family. BMC Evol Biol 9: 85. 19400936 10.1186/1471-2148-9-85 PMC2686710

[17] Seppola M, Johnsen H, Mennen S, Myrnes B, Tveiten H (2009) Maternal transfer and transcriptional onset of immune genes during ontogenesis in Atlantic cod. Dev Comp Immunol 33: 1205–1211. 19577592 10.1016/j.dci.2009.06.013

[18] Chang M, Collet B, Nie P, Lester K, Campbell S, et al (2011) Expression and functional cahracterization of the RIG-I-like receptors MDA5 and LGP2 in rainbow trout *Oncorhynchus mykiss* . J Virol 85: 8403–8412. 21680521 10.1128/JVI.00445-10 PMC3147945

[19] Huang T, Su J, Heng J, Dong J, Zhang R, et al (2010) Identification and expression profiling analysis of grass carp *Ctenophartngodon idella* LGP2 cDNA. Fish Shellfish Immunol 29: 349–355. 20420913 10.1016/j.fsi.2010.04.001

[20] Kasai H, Yoshimizu M (2001) Establishment of Japanese flounder embryo cell lines. Fisheries Sci Hokkaido Univ 52: 67–70.

[21] Ohtani M, Hikima J, Hwang SD, Morita T, Suzuki Y, et al (2012) Transcriptional regulation of type I interferon gene expression by interferon regulatory factor-3 in Japanese flounder, *Paralichthys olivaceus* . Dev Comp Immunol 36: 697–706. 22067740 10.1016/j.dci.2011.10.008

[22] Yazawa R, Hirono I, Aoki T (2006) Transgenic zebrafish expressing chicken lysozyme show resistance against bacterial diseases. Transgenic Res 15: 385–391. 16779653 10.1007/s11248-006-0009-0

[23] Rise ML, Hall JR, Rise M, Hori TS, Browne MJ, et al (2010) Impact of asymptomatic nodavirus carrier state and intraperitoneal viral mimic injection on brain transcript expression in Atlantic cod (*Gadus morhua*). Physiol Genomics 42: 266–280. 20442246 10.1152/physiolgenomics.00168.2009

[24] Sun F, Zhang YB, Liu TK, Shi J, Wang B, et al (2011) Fish MITA serves as a mediator for distinct fish IFN gene activation dependent on IRF3 or IRF7. J Immunol 187: 2531–2539. 21795596 10.4049/jimmunol.1100642

[25] Ooi EI, Hirono I, Aoki T (2006) Functional characterisation of the Japanese flounder, *Paralichthys olivaceus*, Mx promoter. Fish Shellfish Immunol 21: 293–304. 16551503 10.1016/j.fsi.2005.12.004

[26] Yasuike M, Kondo H, Hirono I, Aoki T (2011) Identification and characterization of Japanese flounder, *Paralichthys olivaceus* interferon-stimulated gene 15 (Jf-ISG15). Comp Immunol Microbiol Infect Dis 34: 83–91. 20299096 10.1016/j.cimid.2010.02.005

[27] Kawai T, Akira S (2011) Toll-like receptors and their crosstalk with other innate receptors in infection and immunity. Immunity 34: 637–650. 21616434 10.1016/j.immuni.2011.05.006

[28] McCoy CE, Carpenter S, Pålsson-McDermott EM, Gearing LJ, O'Neill LA (2008) Glucocorticoids inhibit IRF3 phosphorylation in response to Toll-like receptor-3 and -4 by targeting TBK1 activation. J Biol Chem 283: 14277–14285. 18356163 10.1074/jbc.M709731200

